# Pigmented odontogenic keratocyst: Report of a rare case and review of the literature

**DOI:** 10.4317/jced.55134

**Published:** 2018-12-01

**Authors:** Carla-Isabelly Rodrigues-Fernandes, Paulo-Rogério-Ferreti Bonan, Elaine-Judite-de Amorim Carvalho, Celeste Sánchez-Romero, Oslei-Paes de Almeida, Danyel-Elias-da Cruz Perez

**Affiliations:** 1 DDS, MSc student, Piracicaba Dental School, Oral Pathology Area, State University of Campinas, Piracicaba, São Paulo, Brazil; 2 DDS, PhD, School of Dentistry, Stomatology Unit, Universidade Federal da Paraíba, João Pessoa, Paraíba, Brazil; 3 DDS, PhD, Professor, School of Dentistry, Oral Pathology Unit, Universidade Federal de Pernambuco, Recife, Pernambuco, Brazil; 4 DDS, PhD student, Piracicaba Dental School, Oral Pathology Area, State University of Campinas, Piracicaba, São Paulo, Brazil; 5 DDS, PhD, Professor, Piracicaba Dental School, Oral Pathology Area, State University of Campinas, Piracicaba, São Paulo, Brazil

## Abstract

Pigmented odontogenic keratocyst (OKC) is very rare and its etiology remains uncertain. To the best of our knowledge, only 9 cases of pigmented OKC have been published in English-language literature. This report describes a pigmented OKC in a 14-year-old black male patient. Radiographically, the lesion appeared as a well-circumscribed, unilocular, and radiolucent image. A surgical excision was performed. Histopathological examination revealed an OKC. Additionally, a brownish, sparsed, intracytoplasmic pigmentation was observed in the basal cell layer, which was positive for Fontana-Masson staining. Immunohistochemistry reactions revealed positive dendritic cells for S-100 protein, HMB45 and Melan A. No clinical and imaging signs of recurrence were observed after 24 months. In conclusion, melanin apparently does not represent a factor for distinct biological behavior in OKC.

** Key words:**Melanin, melanocytes, odontogenic cyst, odontogenic keratocyst, pigmented.

## Introduction

The odontogenic keratocyst (OKC) is an odontogenic cyst with potential to reach a large size, and presents high recurrence rates. Radiographic findings of OKC include uni- or multilocular radiolucency, surrounded by smooth or scalloped margins, usually with sclerotic borders. Microscopically, OKC presents a regular thin cystic epithelial lining composed of stratified squamous epithelium, with a corrugated parakeratinized surface, a well-defined palisading columnar or cuboidal basal cell layer, and subepithelial clefts (1-3).

Pigmented odontogenic lesions are rare and their etiology remains uncertain. In these lesions, melanin is observed within the cytoplasm of the cystic lining epithelium or tumor cells. Calcifying odontogenic cyst is the odontogenic lesion most commonly associated with pigmentation (4). Pigmented OKC is very rare, with only 9 cases previously published in the English-language literature (4-9). Thus, the purpose of this study was to report a case of pigmented OKC and discuss the hypotheses for this uncommon manifestation.

## Case Report

**Figure 1:**
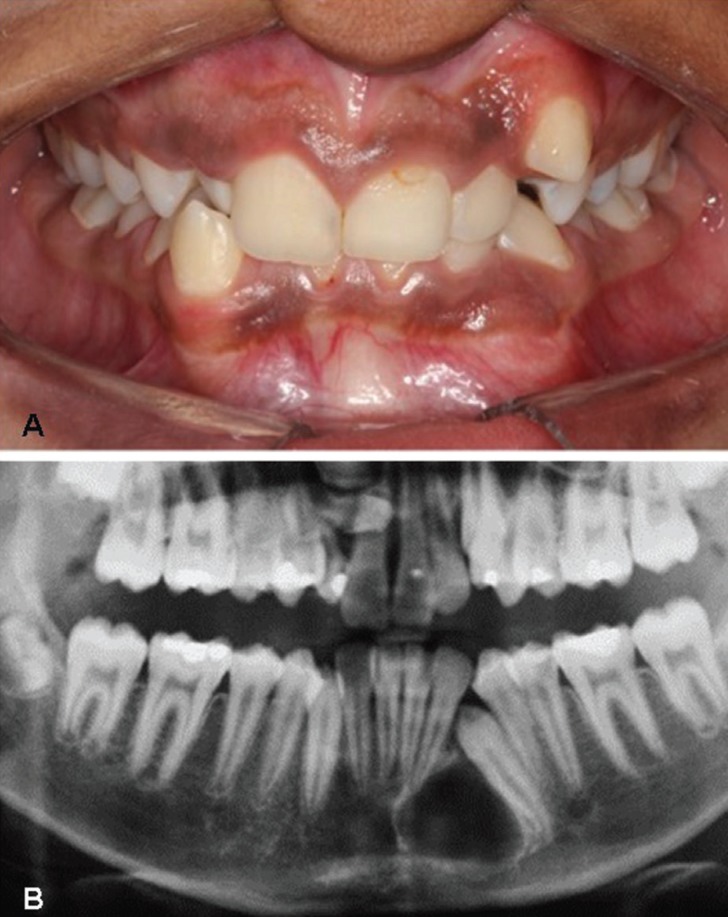
A, Intraoral examination revealed a slight swelling in the left mandibular body, which was covered by normal mucosa. B, Panoramic radiograph showing a well-circumscribed radiolucent lesion displacing the roots of the mandibular left lateral incisor and non-erupted canine.

The patient, a 14-year-old black male was referred for diagnosis of a painless lesion located in the anterior mandibular region. His family could not determine the duration of the lesion. The patient had good general health and absence of extraoral changes. Intraoral examination revealed a painless swelling in the mandibular incisor region, which was covered by intact mucosa with normal color (Fig. 1).

Radiographically, the lesion appeared as an unilocular, radiolucent image, with well-defined borders and sclerotic margins. The lesion also caused divergence of the roots of the mandibular left lateral incisor and canine, which was non-erupted (Fig. 1). OKC and central giant cell lesion were the main diagnostic hypotheses.

**Figure 2:**
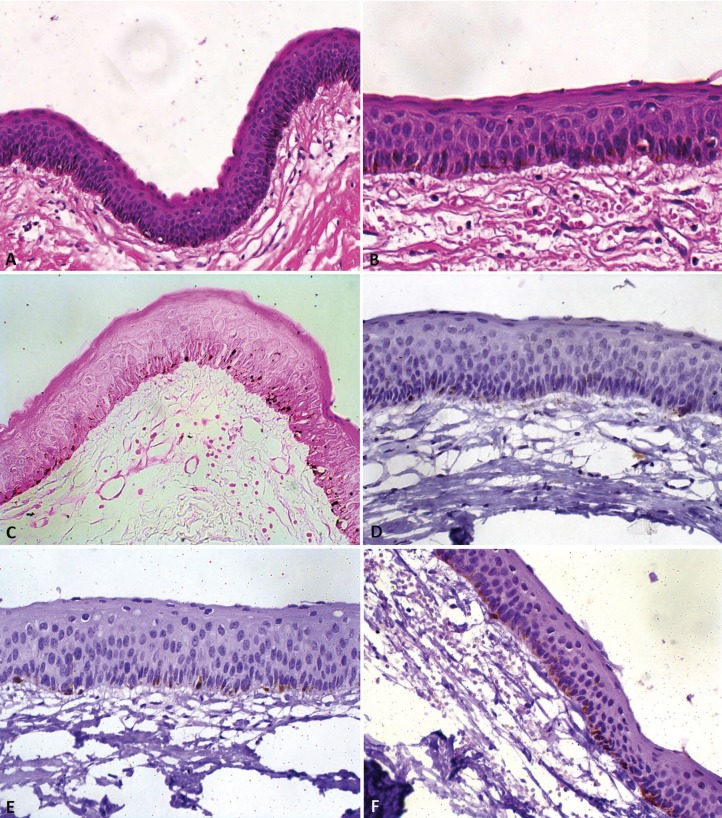
A, Epithelial lining showing corrugated parakeratin, palisading basal cell layer and intracytoplasmic dark brown pigment mainly in basal keratinocytes (hematoxylin-eosin, x100). B, Intracytoplasmic pigment in high-power field (hematoxylin-eosin, x200). C, Intracytoplasmic pigment positive for Fontana-Masson staining (Fontana-Masson, x200). D, Dendritic cells in basal layer positive for S-100 protein (x200). E, Dendritic cells positive for Melan-A, localized in basal cell layer (x200). F, Dendritic cells positive for HMB-45 in basal cell layer (x200).

Under local anesthesia, an excision was performed, due to the notable plane of cleavage when the whole lesion was detached from mandibular bone by means of vigorous curettage. During the surgical procedure, a white-colored material, similar to keratin, was noted, strongly suggestive of OKC. The left mandibular canine was also removed. Histopathological examination revealed a cystic lesion, lined with parakeratinised, stratified, squamous epithelium. The parakeratin appeared corrugated and the basal cell layer showed a palisade arrangement. The fibrous capsule did not present any inflammatory reaction. Additionally, a sparse, brownish, intracytoplasmic pigmentation was observed in the epithelial cells, mainly in the basal layer (Fig. 2). The histopathological diagnosis was OKC. However, the intracytoplasmic pigmentation was further investigated.

The intracytoplasmic pigment was positive for Fontana-Masson staining. Immunohistochemistry reactions showed dendritic cells positive for S-100 protein (polyclonal, dilution 1:10,000), HMB45 (clone HMB45, dilution 1:200), and Melan A (clone A103, dilution 1:800), all localized in the basal cell layer. These findings confirmed the presence of melanocytes and melanin in the cystic epithelial lining. Thus, the final diagnosis was pigmented OKC.

Currently, the patient is under periodic follow up, and no clinical and imaging signs of recurrence have been observed 24 months after the surgical procedure, with complete bone repair.

## Discussion

Pigmented odontogenic lesions are rare but this finding has been observed in a wide variety of lesions, such as calcifying odontogenic cyst, odontogenic keratocyst, adenomatoid odontogenic tumor, ameloblastic fibro-odontoma, complex odontoma, odonto-ameloblastoma, ameloblastic fibroma, odontogenic fibroma, ameloblastic fibro-dentinoma, dentigerous cyst, lateral periodontal cyst, botryoid odontogenic cyst, malignant ameloblastoma, ameloblastic carcinoma, and intraosseous primary carcinoma (4-16).

From a total of 50 cases of pigmented odontogenic lesions published in the English-language literature, only 9 were OKC, which included 6 Japanese patients, 1 white British, 1 black American and 1 West Indian patient (6,7,9,14,15). The pigmented OKCs, including the current case, apparently showed a higher predilection for young patients, with a mean age of 17.5 years (ranging from 11 to 26 years). Six cases occurred in females and 4 in male patients. Two cases presented non-syndromic multiple OKCs (6,15) and one reported the case of a patient with nevoid basal cell carcinoma syndrome (6). The main clinical features of pigmented OKCs are summarized in  Table 1.

**Table 1:**
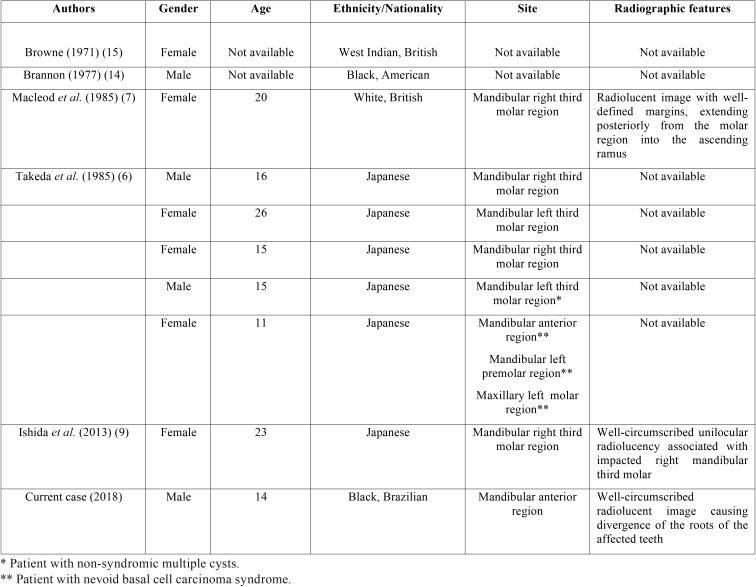
Clinical features of pigmented odontogenic keratocysts reported in the English-language literature.

Relative to the special staining, two studies used Fontana-Masson staining to determine the presence of melanin (6,7), similar to the present case. A case series concluded that the intracytoplasmic pigmentation in an OKC was melanin based on hematoxylin-eosin staining only, and no additional procedure to determine the presence of melanocytes was performed (14). Another case utilized electron microscopy to identify melanocytes (7). Only one study, apart from the present study, performed immunochemistry reactions, in which positive dendritic cells for S100, HMB45 and Melan A were observed in the cystic epithelial lining (9), mainly in basal cell layer. This immunohistochemical profile was also observed in our case.

Several authors have discussed the occurrence of melanin and melanocytes in odontogenic lesions, whose origin remains speculative. The neural crest comprises a diversity of cells, which includes ganglion cells, melanocytes, odontoblasts, and choroidal cells. The movement of these cells has been demonstrated as a balance of active movement and passive displacement, and their subsequent course of differentiation is a product of the synchrony between molecular activity and interactions with the surrounding cells and the environment (17).

The dental lamina originates from the primitive oral lining and the neural crest cells play an important role in odontogenesis as reﬂected in the reciprocal induction that occurs between inner enamel epithelium and cells of the dental papilla, which originate from ectomesenchyme. Differentiation of melanocytes in this neural crest cell-rich zone is thus possible (18). Moreover, Lawson *et al.* (19) observed the presence of melanocytes within the dental lamina and tooth buds of human fetuses, with 12 to 18 weeks, and showed that mucosal melanocytes have a tendency to accumulate around the attachment zone of the dental lamina to the oral epithelium. This suggested the dual role of neural crest cells in melanocyte differentiation, as well as in the formation of the tooth anlage (18-20). Thus, it is considered that melanocytes are present at the base of the dental lamina, and occasionally, may become part of some odontogenic lesions (4-7,20,21), including the OKC.

Other authors discussed that tumor or cystic odontogenic tissue could have potential for neuroectodermal differentiation and then stimulate the formation of melanocytes (4,5). Particularly in OKC, these inactive melanocytes present in the cystic epithelial lining could be activated under unknown circumstances and produce melanin (4-6). Another study also considered that the presence of melanocytes in odontogenic lesions was due to the migration of such cells through the mesenchyme rather than the ectoderm (12). Additionally, some studies considered the association between ethnicity and pigmented OKC, especially due to the number of cases reported in Japanese patients (6,9,18).

Information with regard to treatment, prognosis and follow up of pigmented OKC was mentioned in one previous case only, in which surgical enucleation was performed. The recovery was uneventful and postoperative radiographs demonstrated bone regeneration (7), as demonstrated in this present case. The absence of detailed clinical and follow up information in previous cases does not permit an accurate evaluation about clinical behavior of pigmented OKCs (6,9,14,15). Thus, apart from the few cases previously reported, it remains difficult to assert whether there are behavioral differences between pigmented and non-pigmented OKC.

In conclusion, pigmented OKC is a rare finding and based on this case, the presence of melanocytes apparently did not represent a factor for distinct biological behavior. However, given this peculiar presentation, further investigation about etiology and behavior is necessary to elucidate the theories involving pigmented OKC and other pigmented odontogenic lesions.
